# Draft Genome Sequence of Halomonas hydrothermalis MTCC 5445, Isolated from the West Coast of India

**DOI:** 10.1128/genomeA.01419-14

**Published:** 2015-01-15

**Authors:** Vamsi Bharadwaj SV, Anupama Shrivastav, Sonam Dubey, Tonmoy Ghosh, Chetan Paliwal, Rahulkumar Maurya, Sandhya Mishra

**Affiliations:** a Discipline of Salt & Marine Chemicals, CSIR-CSMCRI, Bhavnagar, Gujarat, India; b Academy of Scientific and Innovative Research (AcSIR), CSIR-CSMCRI, Bhavnagar, Gujarat, India

## Abstract

We announce here the draft genome sequence of Halomonas hydrothermalis MTCC 5445, a halophilic bacterium of the class Gammaproteobacteria. It was isolated from the sea coast of Aadri, Veraval, Gujarat, India. Its genome contains genes for polyhydroxybutyrate (PHB), a biodegradable polymer that can be used as a substitute for petroleum plastics.

## GENOME ANNOUNCEMENT

Halomonas hydrothermalis MTCC 5445 (= SMP3M) was isolated at Aadri, Veraval, Gujarat, India (20°57.584′N 70°16.659′E) (1). It is a Gram-negative, heterotrophic, halophilic, motile organism belonging to the class Gammaproteobacteria and is capable of growing well in salt (NaCl) concentrations up to 5%. H. hydrothermalis has been reported to accumulate polyhydroxybutyrate (PHB) intracellularly at about 75% of its dry cell weight (2). Moreover, it has the ability to utilize a wide variety of carbon sources, including waste glycerol, from biodiesel manufacturing processes for its growth, as well as to produce PHB (1, 3). It is able to ferment sugars, such as maltose, fructose, glucose, sucrose, and ribose. Experiments with seaweed-derived crude levulinic acid established its utility as a cofeed for the bacterium, along with other carbon sources (4). We have also tested Council of Scientific and Industrial Research-Central Salt and Marine Chemicals Research Institute (CSIR-CSMCRI) dry sea mixture as a substitute for commercially available marine bacterial media for its growth (5).

The isolate was deposited at the Microbial Type Culture Collection (MTCC), Institute of Microbial Technology (IMTECH), Chandigarh, India (1), identified as H. hydrothermalis, and given an accession number, MTCC 5445. The 16S rRNA sequence was submitted to GenBank (accession no. GU938192).

The microorganism, by virtue of its salt tolerance, high growth rate, and ability to consume a variety of substrates, is fit for the industrial production of polyhydroxyalkanoate (PHA).

The genome was sequenced at Anand Agricultural University, Anand, Gujarat, India, using a 454-GS FLX sequencer (6). The reads were assembled *de novo* using Newbler version 2.9. The quality analysis of the assembly was performed using QUAST (7). The assembly contained 3,848,774 nucleotides in 64 contigs of >500 bp and 33 contigs of >1,000 bp. A total of 3,467 protein-coding and 63 RNA-coding genes were observed.

The assembled contigs were submitted to the RAST annotation server for subsystem classification and functional annotation (8, 9). The protein-coding genes (CDSs) were assigned using BLASTp with the KEGG Orthology (KO) database.

The draft genome has a mean G+C content of 60.25% and 486 subsystems. Genome analysis revealed genes related to PHA production, which is in agreement with previous reports (1).

The whole-genome information is expected to aid in the metabolic engineering efforts directed toward increasing the production of commercially important products and by-products. Comparative genomic studies will also help in the analysis of diversity and the survival strategies of the bacterium in saline environments.

### Nucleotide sequence accession numbers.

This whole-genome shotgun project has been deposited at DDBJ/EMBL/GenBank under the accession no. JTDR00000000. The version described in this paper is version JTDR01000000.
